# Relevance of Autophagy Induction by Gastrointestinal Hormones: Focus on the Incretin-Based Drug Target and Glucagon

**DOI:** 10.3389/fphar.2019.00476

**Published:** 2019-05-16

**Authors:** Keizo Kanasaki, Emi Kawakita, Daisuke Koya

**Affiliations:** ^1^Department of Diabetology and Endocrinology, Kanazawa Medical University, Uchinada, Japan; ^2^Division of Anticipatory Molecular Food Science and Technology, Medical Research Institute, Kanazawa Medical University, Uchinada, Japan

**Keywords:** autophagy, incretin, GLP-1, DPP-4, glucagon

## Abstract

The biology of autophagy in health and disease conditions has been intensively analyzed for decades. Several potential interventions can induce autophagy in preclinical research; however, none of these interventions are ready for translation to clinical practice yet. The topic of the current review is the molecular regulation of autophagy by glucagon, glucagon-like peptide (GLP)-1 and the GLP-1-degrading enzyme dipeptidyl peptidase-4 (DPP-4). Glucagon is a well-known polypeptide that induces autophagy. In contrast, GLP-1 has been shown to inhibit glucagon secretion; GLP-1 also has been related to the induction of autophagy. DPP-4 inhibitors can induce autophagy in a GLP-1–dependent manner, but other diverse effects could be relevant. Here, we analyze the distinct molecular regulation of autophagy by glucagon, GLP-1, and DPP-4 inhibitors. Additionally, the potential contribution to autophagy by glucagon and GLP-1 after bariatric surgery is discussed.

## Introduction

Recent advances with incretin-based drugs have opened new avenues in the management of diabetes. In the clinic, we can prescribe two types of incretin-based drugs: glucagon-like peptide 1 receptor agonists (GLP-1RAs) and dipeptidyl peptidase-4 (DPP-4) inhibitors. GLP-1 is produced from intestinal L-cells by proteolytical processing from proglucagon (ProG) and immediately degraded by DPP-4; its half-life is approximately 2 min. GLP-1RAs have been developed to avoid DPP-4-mediated cleavage of GLP-1 by introducing a mutation in the amino acid residue that DPP-4 targets. Exenatide (Exendin-4), a 39-amino-acid polypeptide isolated from the venom of the Gila monster lizard with 50% homology to human GLP-1, has been used in the clinic. Alternatively, to extend the half-life of endogenous GLP-1, DPP-4 inhibitors are prescribed. Recent clinical trials (Marso et al., 2016, 2017; Rosenstock et al., 2019) investigating the safety and efficacy of incretin-based drugs have provided diverse interpretations and provocative intellectual curiosities regarding the biology of incretin hormones and incretin-based drugs, specifically focusing on pleiotropic effects.

Autophagy, the cellular mechanism that promotes cell survival during nutrient depletion, may also be relevant under basal or nutrient excess conditions. This cellular process is specified by the formation of autophagosomes, by which cytosolic components are captured and fused with lysosomes to promote the degradation and/or recycling of its contents. The autophagic process consists of four stages: initiation, nucleation, elongation, and fusion/degradation (Codogno et al., 2011). During nutrient depletion, autophagy can provide essential components for energy production and biosynthesis. However, it also acts in a similar manner by recycling damaged organelles, unnecessary proteins, and foreign substances for the quality maintenance of these intracellular components (Ueno and Komatsu, 2017). In circumstances of nutrient excess, autophagy plays important roles in eliminating unfolded proteins and toxic aggregates and facilitating endoplasmic reticulum (ER) homeostasis. The detailed mechanisms and biology of autophagy are summarized in subsequent sections of this issue.

Autophagy defects in certain diseases have been the subject of extensive research. In addition, liver autophagy defects have been shown to occur with several metabolic diseases, such as obesity, steatosis, and type 2 diabetes (Ueno and Komatsu, 2017). Early work in liver research indicates a link in the regulation between gastrointestinal hormones and liver autophagy (Ueno and Komatsu, 2017). Interestingly, incretin hormones and DPP-4 inhibitors have been associated with the amelioration of steatosis (Rowlands et al., 2018; Zheng et al., 2018). These drugs have been shown to induce autophagy in various cell types (Murase et al., 2015; Rowlands et al., 2018; Zheng et al., 2018).

In this review, we investigated the potential involvement of autophagy induction by GLP-1 and incretin-based dugs. In addition, we focused on glucagon, a known polypeptide that regulates glucose levels and a classic molecule that induces autophagy.

## Glucagon and GLP-1 Synthesis From Proglucagon

**Figure 1:**
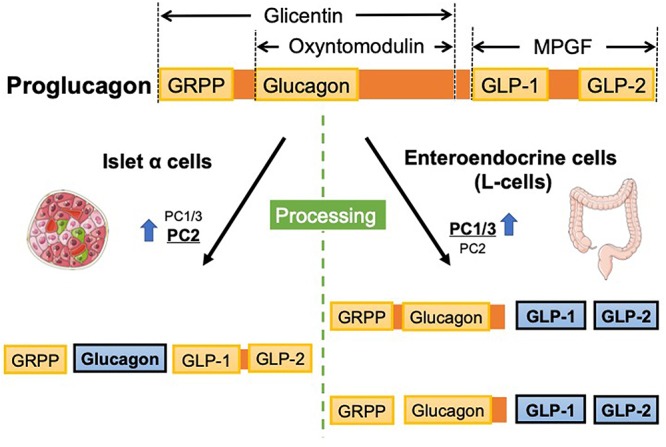
Proglucagon gene (*Gcg*) encodes proglucagon, which is processed in a tissue-specific manner. In pancreatic α cells, glucagon is synthesized from proglucagon because PC2 is dominantly expressed. In contrast, enteroendocrine cells dominantly express PC1/3, ultimately synthesizing GLPs. GRPP, glicentin-related pancreatic peptide; GLP, glucagon-like peptide; MPGF, major proglucagon fragment; PC, prohormone convertase.

GLP-1 is produced from proteolytic cleavage of the precursor polypeptide pProG (Muller et al., 2017). The *pProG* gene (*Gcg*) is expressed in a specific population of enteroendocrine cells (L-cells) in the intestinal mucosa, islet cells in the pancreas, and some neurons within the nucleus of the solitary tract (NTS) (Han et al., 1986; Jin et al., 1988). Regulation of the *Gcg* transcription process is not completely known and distinct pattern of mRNA expression has been reported in intestinal endocrine cells and in pancreatic islet α-cells (Jin, 2008; Yi et al., 2008; Chiang et al., 2012; Muller et al., 2017). In addition to such unique transcriptional control in each cell type, posttranslational processing of prohormone plays an important role in the major cell types producing ProG peptides. In addition to glucagon and GLP-1, glucagon-like peptide-2 (GLP-2), oxyntomodulin, glicentin, glicentin-related pancreatic polypeptide (GRPP), and major proglucagon fragment (MPGF) are synthesized from ProG; however, the specific biological function of some of these fragments has not been identified (Figure 1). Such posttranslational regulation of these ProG peptides in their respective cell types relies on tissue-specific posttranslational modification by prohormone convertases (PCs). In intestinal L-cells and neurons of the NTS, a predominance of PC1/3 expression, GLP-1, oxyntomodulin, and GLP-2 are seen as physiologically relevant (Tucker et al., 1996; Larsen et al., 1997; Vrang et al., 2007); in pancreatic α-cells, high PC2 levels are responsible for the predominant glucagon synthesis (Figure 1) (Holst et al., 1994). PC2 is also expressed in the brain but does not colocalize with *Gcg*. Additionally, PC1/3 is expressed in α-cells but at lower levels than PC2, and the ratio of GLP-1 to glucagon expressed in islet cells has been shown to be increased during the progression of diabetes (O’Malley et al., 2014). *Gcg* expression and ProG levels are relatively lower in the proximal gut and higher in the distal part, with the highest expression in the colon (Bryant and Bloom, 1979).

## Glucagon and Autophagy

The association between autophagy and glucagon was reported approximately 50 years ago. In 1955, Christian de Duve reported on acid phosphatase-positive sac-like particles in rat liver cytoplasm (De Duve et al., 1955). Electron microscopy analysis revealed that such particles are surrounded by a lipoprotein membrane (Novikoff et al., 1956) and later showed acid hydrolases in these organelles, which were subsequently named lysosomes.

Secreted glucagon is recognized by the glucagon receptor (a G protein-coupled receptor) on the hepatocyte, subsequently adenyl cyclase-mediated productions of the second messenger cAMP was stimulated. Rise in the intracellular level of cAMP activates protein kinase A (PKA) and inhibits salt-inducible kinases (SIK). PKA phosphorylates Ser133 of cyclic AMP-responsive element-binding protein (CREB) and SIK dephosphorylates Ser171 of CREB-regulated transcription co-activator (CRTC). Ser133-phosphorylated CREB together with CRTC upregulates CREB target genes such as the gluconeogenesis-related genes PGC1α, nuclear receptor subfamily 4 group A member 1 (NR4A1) and TFEB which regulates gene expressions of autophagy proteins (Ueno and Komatsu, 2017).

In 1962, seminal work by Ashford and Porter (1962) found that glucagon administration increased the autophagy in liver. Subsequently, the role of glucagon in hepatocyte autophagy induction was confirmed *in vivo* by studies in rats (Arstila and Trump, 1968; Guder et al., 1970; Deter, 1971). Such effects of glucagon on the autophagy are likely tissue specific manner (Mortimore and Poso, 1987). Glucagon could induce autophagy by increasing the size and number of autophagic vacuoles (Guder et al., 1970; Deter, 1971; Shelburne et al., 1973); in addition, glucagon enhanced the fragility of hepatic lysosomes both mechanically and osmotically and altered sedimentation properties (Deter and De Duve, 1967). Such effects of glucagon on the hepatic lysosome appeared 30 min after intraperitoneal administration of glucagon, peaked for 15–30 min, and disappeared after approximately 4 h (Deter and De Duve, 1967). The number of hepatic lysosomes increased under conditions associated with an increase in endogenous glucagon levels, such as starvation (Guder et al., 1970), hypoglycemia induced by phlorizin (Becker and CornwallJr., 1971), or type 1 diabetes (Amherdt et al., 1974). Supporting these findings, a significant correlation between the parameters of hepatic lysosomal volume density and plasma glucagon was observed in rats with type 1 diabetes induced by streptozotocin, and insulin intervention in these rats led to suppression of glucose and glucagon levels (Amherdt et al., 1974). In addition, pancreatic transplantation normalized liver autophagy levels in rats with streptozotocin-induced diabetes by restoring insulin and glucagon levels (Brekke et al., 1983). Glucagon is relevant to glucagon-mediated glycogenolysis; glycogen granules are selectively enveloped by autophagosomes for catabolism into glucose. This special type of autophagy is termed glycophagy.

## GLP-1-Related Autophagy

GLP-1RA has been shown to suppress glucagon levels (Mentis et al., 2011). Even though tissue diversity effects of glucagon on the autophagy induction, the liver is the established target organ for glucagon-induced autophagy; therefore, from this point of view, GLP-1 signaling could be relevant to inhibiting autophagy induction in liver. Recently, however, GLP-1 has also been implicated in the induction of autophagy in the liver (He et al., 2016) and in β cells (Zummo et al., 2017; Arden, 2018) as well.

GLP-1 can protect β cells from insults induced by chronic exposure to excess nutrients via induction of autophagosomal-lysosomal fusion (Zummo et al., 2017; Arden, 2018). Exendin-4, an agonistic polypeptide for human GLP-1R derived from the venom of the Gila monster lizard, has also been shown to enhance lysosomal function in β-cells, improve autophagosome clearance and protect against islet injury in a rat model of tacrolimus-induced diabetes (Lim et al., 2016). Indeed, in this study, β cells from rats administered Exendin-4 showed a reduction in the number of autophagosomes (Lim et al., 2016). Therefore, in certain environments, contrary to the hypothesis of the antiglucagon and antiautophagic signaling effects of GLP-1, GLP-1 receptor signaling could be relevant to the accelerated effects on autophagosomal-lysosomal fusion and the positive mediation of autophagic flux. However, the role of GLP-1 in β-cell autophagy is complex and likely dependent on stress conditions. In a rat model fed with high levels of fructose, GLP-1 analog intervention induced notable inhibition of β cell autophagy and enhanced β cell mass and function (Maiztegui et al., 2017). The detailed molecular regulation of the autophagy system in β cells via GLP-1 receptor signaling requires further investigation.

**Figure 2:**
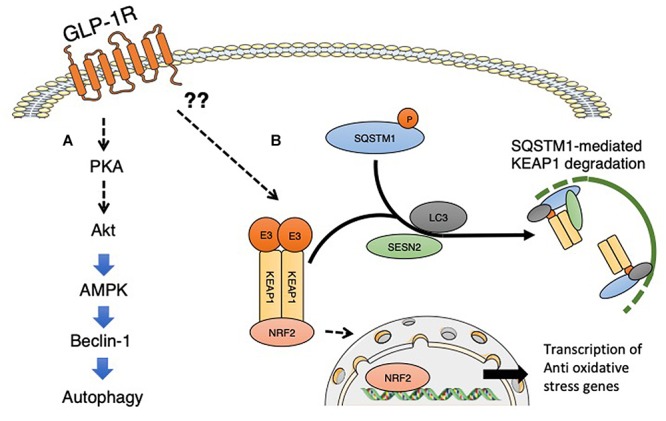
GLP-1 receptor signaling and induction of autophagy. **(A)** GLP-1 receptor signaling activated PKA signaling, and subsequent activation of Akt and AMPK induced the proautophagic molecule Beclin-1. **(B)** GLP-1 receptor signaling is associated with NFR2 activation and the subsequent induction of oxidative stress-responsive genes. Even though detailed mechanisms have not yet been elucidated, GLP-1 signaling could potentially induce the interaction between SQSTM1 and KEAP1, resulting in the suppression of KEAP1-driven ubiquitination of NRF2; phosphorylated SQSTM1 and KEAP1 complexes are selectively degraded by autophagy. The involvement of sestrin2 associated with GLP-1 receptor signaling has not yet been analyzed. SESN2, sestrin2.

The liver is the potential target organ for GLP-1-induced autophagy (He et al., 2016). Among the organs, the level of GLP-1 in the liver is highest because of transport through the hepatic portal vein from the gut. Intervention of GLP-1 or its analogs could ameliorate several aspects of liver injury (D’Alessio et al., 2004; Cantini et al., 2016) and influence hepatic gluconeogenesis, glycogen synthesis, and glycolysis (Figure 2). For the effects of GLP-1, its receptor GLP-1R is essential; the presence of GLP-1R is controversial in hepatocytes and the focus of intense discussion. Protein expression of GLP-1R has been reported in transformed human hepatocyte cell lines, Hep-G2, HuH7, and primary human hepatocytes (Gupta et al., 2010). Even though there is confirmation of GLP-1R expression on hepatocytes, research has suggested that some effects of GLP-1 are indeed GLP-1R-independent events (Bullock et al., 1996; Flock et al., 2007; Aviv et al., 2009; Tomas et al., 2010). An alternative possible explanation could be based on the GLP-1 degradation products, such as GLP-1 9-36, GLP-1 28-36, or GLP-1 32-36. Studies have indicated that both GLP-1 28–36 and GLP-1 32–36 are cell-penetrating peptides that do not require a GLP-1R (Elahi et al., 2014). GLP-1(32–36) amide, a novel pentapeptide cleavage product of GLP-1, modulates whole-body glucose metabolism in dogs (Elahi et al., 2014). GLP1-derived nonapeptide GLP1(28–36) amide preserves pancreatic β cells from glucolipotoxicity (Liu et al., 2012) and activates PKA and Wnt signaling [reviewed in (Jin and Weng, 2016)]. The beneficial effects of GLP-1 fragments were reported to include kidney protective effects in *db/db* mice with diabetes (Moellmann et al., 2018). Whether such GLP-1 derived fragments are relevant for the GLP-1-induced liver autophagy induction is not known and required further investigation.

A series of experiments provided evidence that a GLP-1 agonist ameliorated hepatic steatosis and metabolic defects. Such antisteatotic and metabolic effects of GLP-1 on the liver could involve AMPK activation and suppression of the mTOR pathway. Liraglutide has been shown to halt the progression of steatosis and is associated with the induction of autophagy via activation of AMPK and suppression of mTOR pathways (He et al., 2016). By activating both macro- and chaperone-mediated autophagy, GLP-1 could protect hepatocytes from fatty acid-related apoptosis by suppressing a dysfunctional ER stress response. GLP-1 therapies have been shown to relieve the burden on the ER, reduce ER stress, and decrease subsequent hepatocyte apoptosis (Sharma et al., 2011).

The alternative explanation of autophagy induction by GLP-1 could be associated with suppression of oxidative stress. GLP-1 agonists have been shown to reduce oxidative stress in diverse preclinical studies. When the endogenous antioxidant system cannot remove free radicals appropriately, oxidative stress, the accumulation of such free radicals, accelerates a variety of disease conditions, such as diabetes and its complications, cancer, and neuronal disorders. Cellular defense systems for combatting reactive oxygen species (ROS) rely on the presence of antioxidants that scavenge ROS or induce genes involved in cytoprotection to neutralize ROS (Kaspar et al., 2009; Ma, 2013). The nuclear factor erythroid 2-related factor 2 (Nrf2) transcription factor is a master regulator of redox balance and is responsible for the transcription of various antioxidant and detoxification genes by binding to antioxidant response elements (AREs) (Kaspar et al., 2009; Ma, 2013). GLP-1 agonists have been shown to induce Nrf2 in β cells (Oh and Jun, 2017).

When cells are exposed to oxidative stress, SQSTM1, known as the ubiquitin-binding protein p62 and an autophagosome cargo protein, is phosphorylated at Ser349. Phosphorylated form of SQSTM1 physically interacted with KEAP1, an adaptor of the ubiquitin ligase complex for Nrf2, with high affinity (Ueno and Komatsu, 2017). The interaction between SQSTM1 and KEAP1 results in the suppression of KEAP1-driven ubiquitination of Nrf2; phosphorylated SQSTM1 and KEAP1 complexes are selectively degraded by autophagy (Ueno and Komatsu, 2017). Thereafter, Nrf2 is stabilized, translocates into the nucleus, and induces the expression of various essential cytoprotective genes, such as NAD(P)H dehydrogenase quinone 1, glutathione *S*-transferase, glutamate-cysteine ligase catalytic subunit and heme oxygenase 1 (Jain et al., 2010; Komatsu et al., 2010; Lau et al., 2010; Taguchi et al., 2012; Ichimura et al., 2013). Sestrin 2, also known as an intracellular leucine sensor that negatively regulates mTORC1 signaling, binds with the SQSTM1 and KEAP1 complexes and functions as a scaffold protein for the SQSTM1-mediated autophagy of KEAP1 (Bae et al., 2013). Sestrin 2 is also induced under conditions of stress (Yang et al., 2014); Nrf2 activation might be regulated by selective autophagy under metabolic stress. Therefore, GLP-1-induced Nfr2 activation could be relevant to GLP-1-induced autophagy, but further study is needed. The association between GLP-1 and sestrin 2 has yet to be confirmed.

## DPP-4 Inhibitors-Induced Autophagy

DPP-4, a member of the serine peptidase/prolyl oligopeptidase gene family, was first found as a T cell differentiation antigen (CD26) and also as cell surface aminopeptidase. DPP-4 displays numerous biological functions, such as protease activity, interaction with adenosine deaminase and the extracellular matrix proteins, co-receptor activity mediating viral entry, and regulation of intracellular signals (Kameoka et al., 1993; Kahne et al., 1999; Lambeir et al., 2003; Lopez-Otin and Matrisian, 2007; Lu et al., 2013). Furthermore, the complexities of the biological functions of DPP-4 are indeed multiplying with diverse bioactive substrates of DPP-4, thus emphasizing the elegant role of DPP-4 in the biochemical tuning of multiple cell type and tissues. DPP-4 inhibitors exhibited multiple organ protective potential (Kroller-Schon et al., 2012; Itou et al., 2013; Kanasaki, 2016; Zhuge et al., 2016; Avogaro and Fadini, 2018) and also influenced cancer biology (Abrahami et al., 2018; Ye et al., 2018; Enz et al., 2019; Hollande et al., 2019; Yang et al., 2019).

Some preclinical studies have shown a potential link between DPP-4 inhibition and autophagy induction. In leptin-deficient *ob/ob* mice, sitagliptin at 50 mg/kg daily for 4 weeks ameliorated weight gain, metabolic disorders, and steatosis in the liver as well as insulin sensitivity. In this study, sitagliptin increased AMPK phosphorylation and decreased mTOR phosphorylation associated with the restoration of ATG5 and Beclin 1 messenger RNA expression that was suppressed in *ob/ob* mice. In addition, the relative level of LC3-II/LC3-I was significantly diminished in *ob/ob* mice and was restored to the basal level by sitagliptin (Zheng et al., 2018). Another report showed that autophagic responses were significantly diminished in OLETF rats after experimental myocardial infarction associated with a deficiency in AMPK/ULK-1 activation, Akt/mTOR/S6 signaling, and increased Beclin-1-Bcl-2 interaction, which are key molecular events for suppressing autophagy. Intervention with the DPP-4 inhibitor vildagliptin inhibited the Beclin-1-Bcl-2 interaction and enhanced both LC3-II protein and autophagosomes in the non-infarcted region in OLETF rats without normalization of either AMPK/ULK-1 or mTOR/S6 signaling. Such effects of vildagliptin on heart autophagy are associated with an 80% survival rate in OLETF rats; chloroquine, an autophagy inhibitor, diminished these beneficial effects of vildagliptin (Murase et al., 2015).

Reports have indicated that DPP-4 inhibitors could be associated with the induction of autophagy; however, the underlying mechanisms by which DPP-4 inhibition is related to autophagy induction are not absolutely clear. It may involve an increase in levels of GLP-1 by DPP-4 inhibitor treatment. DPP-4 inhibitor enhanced insulin secretion and induction of autophagy signals in the islets of high-fat-fed mice; such effects of DPP-4 inhibition on autophagy signaling were completely abolished by GLP-1R antagonist exendin 9-39 coadministration (Liu et al., 2016).

## DPP-4 and Extracellular Matrix Interaction: Relevance to Autophagy Suppression

Other than GLP-1 induction, the pleiotropic effects of a DPP-4 inhibitor may be relevant to the mechanisms of autophagy induction by DPP-4 inhibition.

The interaction with the extracellular matrix is an important determinant of cell fate. Integrins are glycoproteins that play vital roles in cell–cell or cell–matrix interactions through αβ heterodimers. Eighteen α and eight β subunits of integrins are known, and each of them displays diverse ligand binding and signaling properties (Pozzi and Zent, 2011). Integrin subunits consist of an extracellular domain that is important for their ligand binding properties and contains a transmembrane domain and a short cytoplasmic tail, which could interact with diverse cytosolic and transmembrane proteins by consisting a focal adhesion complex (with the exception of β4) (Pozzi and Zent, 2003). Integrins display physical interaction with several extracellular matrix (ECM) glycoproteins (such as collagens, fibronectins, and laminins) and cellular receptors (Plow et al., 2000; Hynes, 2002). Integrins are essential molecules in actin cytoskeleton remodeling and in regulating cell signals that regulate biological and cellular functions (Park et al., 2015). Integrins display intracellular signaling through ligand binding (“outside-in” signaling) (Ratnikov et al., 2005). Alternatively, integrins can alter their high- to low-affinity conformations, facilitating specific ligand binding (“inside-out” signaling) (Luo et al., 2007). The activation status of integrin relies on the cell type. In most cells that adhere to the basement membrane, integrins are activated; in contrast, integrins are inactive in circulating platelets or leukocytes until they are induced by platelet aggregation or stimulated by an inflammatory response. Integrins contain neither a catalytic site nor kinase activity but play a role as a bridge between the ECM and actin cytoskeleton. Such interaction between the ECM and the actin cytoskeleton through integrins allows integrins to maintain cytoskeletal organization, cell motility and intracellular-signaling pathways such as cell survival, cell shape, cell proliferation, and angiogenesis (Arnaout et al., 2007; Luo et al., 2007).

**Figure 3:**
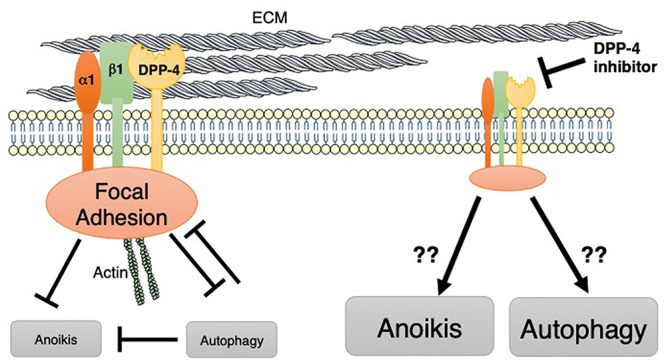
DPP-4 may target integrin β1-mediated suppression of autophagy. On the cell membrane, DPP-4 displays an interaction with integrin β1. Integrin β1 is important for focal adhesion complexes. These integrin β1-mediated cell adhesion complexes potentially inhibit both anoikis and autophagy. Indeed, autophagy is an important biological mechanism for protecting cells from anoikis as well. Autophagy also targets and inhibits focal adhesion complexes.

Indeed, DPP-4 is the molecule that interacts with the key integrin, integrin β1 (Figure 3), which can form a heterodimer with at least 11 α-subunits. Integrin β1 has the biological function of a “hub integrin” and acts as a receptor for specific ECM components, revealed in kidney epithelial cells (Glynne et al., 2001) or in T cell lymphoma (Elias et al., 2014). The loss of membrane-bound DPP-4 has been associated with suppression of the phosphorylation of integrin β1 S785, which plays a key role in the cellular adhesion of integrin β1 to the ECM (Sato et al., 2005). We have shown that the DPP-4 inhibitor linagliptin suppressed the interaction between DPP-4 and integrin β1, subsequently inhibiting the endothelial to mesenchymal transition program (Shi et al., 2015; Kanasaki, 2016), the fibrogenic programs associated with the inhibition of autophagy (Singh et al., 2015). Interestingly, in addition to the biological importance of integrin β1 on the suppression of autophagy, the autophagy pathway targets integrin β1 during nutrient starvation (Vlahakis and Debnath, 2017). Autophagy degrades focal adhesion proteins and promotes turnover of those molecules (Vlahakis and Debnath, 2017). Additionally, integrin-mediated cell adhesion to the ECM has been shown to protect cells from anoikis, the apoptosis induced by the lack of correct cell/ECM attachment. Once integrin-mediated interaction with the ECM is lost, cells induce autophagy for survival (Figure 3). Autophagy induction has been shown to promote the survival of epithelial cells and adjustments in the absence of cell–matrix contact, resulting in the anoikis resistance (Yang et al., 2013; Chen et al., 2017; Talukdar et al., 2018) (Figure 3). After autophagy was inhibited by either RNA interference or harboring of oncogenes, cells lost their ability to combat anoikis (Figure 3).

## Perspective: Bariatric Surgery and Autophagy Induction

Bariatric surgery, including Roux-en-Y (RYGB), gastric banding, sleeve gastrectomy (SG), and biliopancreatic diversion (BPD), has been a beneficial intervention in the treatment of obesity for reduction in body weight and is associated with the amelioration of liver steatosis and metabolic defects. However, the detailed molecular mechanisms of the beneficial metabolic effects of bariatric surgery have not been completely established.

**Figure 4:**
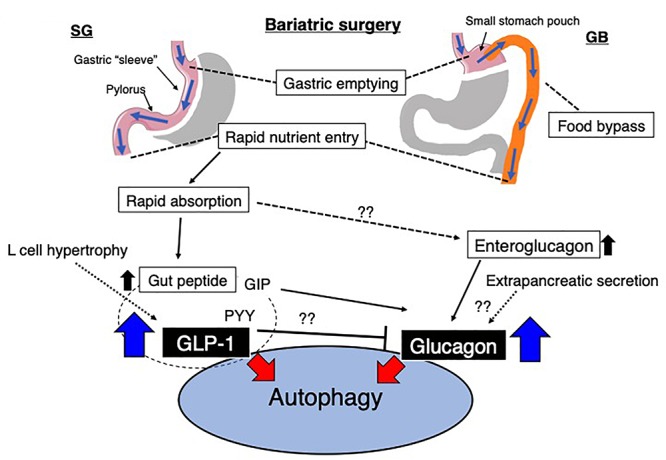
Possible alterations in the gut environment and the relationship to autophagy after bariatric surgery. Sleeve gastrectomy (SG) and Roux-en-Y gastric bypass (GB) are the most common surgical procedures in bariatric surgery. After bariatric surgery, the rapid entry of nutrients likely induces the secretion of GLP-1. Additionally, glucagon is potentially increased by various/unknown mechanisms. GLP-1 and other gut-derived peptides, such as PYY, could inhibit glucagon expression, and GIP might stimulate glucagon expression. However, these observations were all investigated in pancreatic cells, and the regulation of ‘gut glucagon’ was not found. GIP, gastric inhibitory peptide; PYY, peptide YY; GLP-1, glucagon-like peptide-1.

Some reports indicate that amelioration of metabolic profiling by gastrectomy was associated with autophagy induction (Soussi et al., 2016). Several possible explanations were made for this interesting phenomenon. After bariatric surgery, significant alterations in anatomical structure induced changes in the integrated responses during eating, including cephalic phase, chewing and tasting, gastric phase, intestinal phase and gut peptides, absorptive phase, glucose metabolism, liver and bile acid phase, and large intestine and microbiota phase [summarized in ref (Quercia et al., 2014)]. The molecules described in this review could be also notable for their significance in autophagy induction by bariatric surgery (Figure 4).

### Glucagon

In the stomach, gastric emptying is regulated by gastric content and neural and hormonal influences and is altered after bariatric surgery as described earlier. Accelerated gastric emptying time for liquids but slower gastric emptying time for solids have been reported after RYGB (Horowitz et al., 1982). Kotler et al. (1985) reported faster intestinal transit time and increased enteroglucagon levels in patients with greater weight loss compared to weight-stable patients. The term “enteroglucagon” in this report did not necessarily include “glucagon” because of cross reaction with several glucagon sequence-containing polypeptides at the time of this report. The presence of extrapancreatic glucagon secretion in humans has been the focus of intense discussion for years, and even though evidence was conflicting, some investigators reported that glucagon responses after total pancreatectomy were present in animals (Sutherland and De Duve, 1948; Matsuyama and Foa, 1974; Vranic et al., 1974; Muller et al., 1978; Doi et al., 1979; Gotoh et al., 1989) and humans (Unger et al., 1966; Barnes and Bloom, 1976; Villanueva et al., 1976; Boden et al., 1980; Karesen et al., 1980; Sudo et al., 1980; Dammann et al., 1981; Holst et al., 1983; Yasui, 1983; Polonsky et al., 1984; Bajorunas et al., 1986a,b; Ohtsuka et al., 1986; Tanjoh et al., 2003). The most challenging point was that until recently, analytical methods for glucagon have not been sufficiently sensitive or specific to justify decisive statements about the absence or presence of extrapancreatic fully processed 29–amino acid glucagon (Tanjoh et al., 2003). Recently, sandwich enzyme-linked immunosorbent assays (ELISA) utilizing a combination of C- and N-terminal antiglucagon antibodies have been emerged. Such ELISA system theoretically could eliminate cross-reactivity with truncated or elongated forms of glucagon containing polypeptides (Wewer Albrechtsen et al., 2014). Lund et al. (2016) studied patients who underwent total pancreatectomy and analyzed plasma glucagon levels. As expected, the gastrointestinal anatomy was remarkably changed, including the removal of the pyloric sphincter and duodenum after total pancreatectomy. Therefore, following the ingestion of a meal, nutrients are rerouted and delivered directly from the stomach to the jejunum in a manner similar to bariatric surgery as previously described. The unique point of the study by Lund et al. (2016) is that they utilized not only novel sandwich enzyme-linked immunosorbent assays of plasma glucagon but also mass spectrometry-based proteomics to confirm 29-amino acid circulatory glucagon levels in patients without a pancreas. Basal glucagon levels in these patients exhibited a lower trend, and glucose challenge of the gastrointestinal tract exerted significant hyperglucagonemia in these patients. Lund et al. (2016) also confirm that the intravenous glucose infusion attenuated plasma glucagon levels, and directs focus on the gastrointestinal tract. Unfortunately, there is no direct evidence indicated the hyperglucagonemia after bariatric surgery yet, but higher glucagon release within the first 2 h and higher trend of peak level of glucagon in post RYGB patients when compared to SG or neither operation group has been recently reported (Svane et al., 2019). These findings suggest that alteration in glucagon secretion or possibly hyperglucagonemia could be induced by bariatric surgery and be relevant in systemic physiological alterations, including autophagy (Figure 4). Subsequently such induction of autophagy could influence on the health in individuals with obesity but also in lean and in malnourished patients after gastrectomy. Further research would be required in this field.

### GLP-1

The potential contribution of GLP-1 to the pathobiological alteration that occurs after bariatric surgery was seen in adverse outcomes of bariatric surgery. Late dumping syndrome, a well-known hypoglycemic event, occurs 2–3 h after a meal, after gastric surgery, and as a complication after surgery for obesity as well. The underlying cause of late dumping syndrome is not completely understood, but in general, it is hypothesized that postoperative elevation of incretin hormone, especially GLP-1 levels, leads to pancreatic β-cell hypertrophy (Figure 4). Such β-cell hypertrophy stimulates insulin secretion and hypoglycemic symptoms. These theories are supported by the observation that hyperinsulinemic hypoglycemia most frequently occurred in patients who underwent resection of parts of the stomach, in which the gastrojejunostomy bypasses the pylorus. Therefore, induced levels of GLP-1 during an oral glucose or meal test have been consistently reported after RYGB (Yi et al., 2008), BPD (Jin and Weng, 2016), and SG (Moellmann et al., 2018) (Figure 4). Even though none of these theories have been mechanistically proven during a hypoglycemic event, an increase in GLP-1 could be associated with hepatic autophagy. He et al. (2015) reported that in a study of rats with obesity and diabetes, RYGB led to significant induction of autophagy in the liver, restored autophagy levels in the liver and was associated with reducing the level of hepatic lipids. Increased autophagy in the liver after RYGB was well correlated with plasma GLP-1 levels (He et al., 2015). Therefore, induction of GLP-1 after bariatric surgery would also lead to significant stimulation of autophagy (Figure 4).

## Conclusion

In this review, we focused on glucagon/GLP-1 and associated drugs in the physiology of autophagy. We propose that changes in the gastrointestinal tract that induces food to bypass the intestine would be relevant to the induction of autophagy via secretion of GLP-1 and/or glucagon.

## Author Contributions

KK concept, design, and major contribution to writing the manuscript. EK made figures and discussion. DK intellectual input in the projects.

## Conflict of Interest Statement

KK is under a consultancy agreement with Boehringer Ingelheim. The remaining authors declare that the research was conducted in the absence of any commercial or financial relationships that could be construed as a potential conflict of interest.

## References

[B1] Abrahami D. Yin H. Yu O. H. Y. Pollak M. N. Azoulay L. (2018). Incretin-based drugs and the incidence of colorectal cancer in patients with type 2 diabetes. *Epidemiology* 29 246–253. 10.1097/EDE.0000000000000793 29283894

[B2] Amherdt M. Harris V. Renold A. E. Orci L. Unger R. H. (1974). Hepatic autography in uncontrolled experimental diabetes and its relationships to insulin and glucagon. *J. Clin. Invest.* 54 188–193. 10.1172/jci107742 4834889 PMC301539

[B3] Arden C. (2018). A role for glucagon-like peptide-1 in the regulation of beta-cell autophagy. *Peptides* 100 85–93. 10.1016/j.peptides.2017.12.002 29412836

[B4] Arnaout M. A. Goodman S. L. Xiong J. P. (2007). Structure and mechanics of integrin-based cell adhesion. *Curr. Opin. Cell Biol.* 19 495–507. 10.1016/j.ceb.2007.08.002 17928215 PMC2443699

[B5] Arstila A. U. Trump B. F. (1968). Studies on cellular autophagocytosis. The formation of autophagic vacuoles in the liver after glucagon administration. *Am. J. Pathol.* 53 687–733. 4300890 PMC2013521

[B6] Ashford T. P. Porter K. R. (1962). Cytoplasmic components in hepatic cell lysosomes. *J. Cell Biol.* 12 198–202. 10.1083/jcb.12.1.198 13862833 PMC2106008

[B7] Aviv V. Meivar-Levy I. Rachmut I. H. Rubinek T. Mor E. Ferber S. (2009). Exendin-4 promotes liver cell proliferation and enhances the PDX-1-induced liver to pancreas transdifferentiation process. *J. Biol. Chem.* 284 33509–33520. 10.1074/jbc.M109.017608 19755420 PMC2785195

[B8] Avogaro A. Fadini G. P. (2018). The pleiotropic cardiovascular effects of dipeptidyl peptidase-4 inhibitors. *Br. J. Clin. Pharmacol.* 84 1686–1695. 10.1111/bcp.13611 29667232 PMC6046494

[B9] Bae S. H. Sung S. H. Oh S. Y. Lim J. M. Lee S. K. Park Y. N. (2013). Sestrins activate Nrf2 by promoting p62-dependent autophagic degradation of Keap1 and prevent oxidative liver damage. *Cell Metab.* 17 73–84. 10.1016/j.cmet.2012.12.002 23274085

[B10] Bajorunas D. R. Fortner J. G. Jaspan J. B. (1986a). Glucagon immunoreactivity and chromatographic profiles in pancreatectomized humans. Paradoxical response to oral glucose. *Diabetes* 35 886–893. 10.2337/diab.35.8.886 3525286

[B11] Bajorunas D. R. Fortner J. G. Jaspan J. Sherwin R. S. (1986b). Total pancreatectomy increases the metabolic response to glucagon in humans. *J. Clin. Endocrinol. Metab.* 63 439–446. 10.1210/jcem-63-2-439 3522619

[B12] Barnes A. J. Bloom S. R. (1976). Pancreatectomised man: a model for diabetes without glucagon. *Lancet* 1 219–221. 10.1016/s0140-6736(76)91339-8 55531

[B13] Becker F. F. Cornwall C. C. Jr. (1971). Phlorizin induced autophagocytosis during hepatocytic glycogenolysis. *Exp. Mol. Pathol.* 14 103–109. 10.1016/0014-4800(71)90056-6 5540981

[B14] Boden G. Master R. W. Rezvani I. Palmer J. P. Lobe T. E. Owen O. E. (1980). Glucagon deficiency and hyperaminoacidemia after total pancreatectomy. *J. Clin. Invest.* 65 706–716. 10.1172/jci109717 6986412 PMC371413

[B15] Brekke I. B. Danielsen H. Reith A. (1983). Normalization of hepatic lysosomal autophagy in streptozotocin diabetic rats after pancreatic transplantation. *Virchows Arch. B Cell Pathol. Incl. Mol. Pathol.* 43 189–197. 10.1007/bf02932956 6137107

[B16] Bryant M. G. Bloom S. R. (1979). Distribution of the gut hormones in the primate intestinal tract. *Gut* 20 653–659. 10.1136/gut.20.8.653 114457 PMC1412529

[B17] Bullock B. P. Heller R. S. Habener J. F. (1996). Tissue distribution of messenger ribonucleic acid encoding the rat glucagon-like peptide-1 receptor. *Endocrinology* 137 2968–2978. 10.1210/en.137.7.2968 8770921

[B18] Cantini G. Mannucci E. Luconi M. (2016). Perspectives in GLP-1 research: new targets. New receptors. *Trends Endocrinol. Metab.* 27 427–438. 10.1016/j.tem.2016.03.017 27091492

[B19] Chen J. L. David J. Cook-Spaeth D. Casey S. Cohen D. Selvendiran K. (2017). Autophagy induction results in enhanced anoikis resistance in models of peritoneal disease. *Mol. Cancer Res.* 15 26–34. 10.1158/1541-7786.MCR-16-0200-T 27807188 PMC5909689

[B20] Chiang Y. T. Ip W. Jin T. (2012). The role of the Wnt signaling pathway in incretin hormone production and function. *Front. Physiol.* 3:273 10.3389/fphys.2012.00273 PMC3429047 22934027

[B21] Codogno P. Mehrpour M. Proikas-Cezanne T. (2011). Canonical and non-canonical autophagy: variations on a common theme of self-eating? *Nat. Rev. Mol. Cell Biol.* 13 7–12. 10.1038/nrm3249 22166994

[B22] D’Alessio D. Vahl T. Prigeon R. (2004). Effects of glucagon-like peptide 1 on the hepatic glucose metabolism. *Horm. Metab. Res.* 36 837–841. 10.1055/s-2004-826172 15655716

[B23] Dammann H. G. Besterman H. S. Bloom S. R. Schreiber H. W. (1981). Gut-hormone profile in totally pancreatectomised patients. *Gut* 22 103–107. 10.1136/gut.22.2.103 7215941 PMC1419224

[B24] De Duve C. Pressman B. C. Gianetto R. Wattiaux R. Appelmans F. (1955). Tissue fractionation studies. 6. Intracellular distribution patterns of enzymes in rat-liver tissue. *Biochem. J.* 60 604–617. 10.1042/bj0600604 13249955 PMC1216159

[B25] Deter R. L. (1971). Quantitative characterization of dense body, autophagic vacuole, and acid phosphatase-bearing particle populations during the early phases of glucagon-induced autophagy in rat liver. *J. Cell Biol.* 48 473–489. 10.1083/jcb.48.3.473 4322760 PMC2108109

[B26] Deter R. L. De Duve C. (1967). Influence of glucagon, an inducer of cellular autophagy, on some physical properties of rat liver lysosomes. *J. Cell Biol.* 33 437–449. 10.1083/jcb.33.2.437 4292315 PMC2108350

[B27] Doi K. Prentki M. Yip C. Muller W. A. Jeanrenaud B. Vranic M. (1979). Identical biological effects of pancreatic glucagon and a purified moiety of canine gastric immunoreactive glucagon. *J. Clin. Invest.* 63 525–531. 10.1172/jci109331 429572 PMC371982

[B28] Elahi D. Angeli F. S. Vakilipour A. Carlson O. D. Tomas E. Egan J. M. (2014). GLP-1(32-36)amide, a novel pentapeptide cleavage product of GLP-1, modulates whole body glucose metabolism in dogs. *Peptides* 59 20–24. 10.1016/j.peptides.2014.06.004 24937653 PMC5155305

[B29] Elias B. C. Mathew S. Srichai M. B. Palamuttam R. Bulus N. Mernaugh G. (2014). The integrin beta1 subunit regulates paracellular permeability of kidney proximal tubule cells. *J. Biol. Chem.* 289 8532–8544. 10.1074/jbc.M113.526509 24509849 PMC3961677

[B30] Enz N. Vliegen G. De Meester I. Jungraithmayr W. (2019). CD26/DPP4 - a potential biomarker and target for cancer therapy. *Pharmacol. Ther.* [Epub ahead of print]. 30822465 10.1016/j.pharmthera.2019.02.015

[B31] Flock G. Baggio L. L. Longuet C. Drucker D. J. (2007). Incretin receptors for glucagon-like peptide 1 and glucose-dependent insulinotropic polypeptide are essential for the sustained metabolic actions of vildagliptin in mice. *Diabetes Metab. Res. Rev.* 56 3006–3013. 10.2337/db07-0697 17717280

[B32] Glynne P. A. Picot J. Evans T. J. (2001). Coexpressed nitric oxide synthase and apical beta(1) integrins influence tubule cell adhesion after cytokine-induced injury. *J. Am. Soc. Nephrol.* 12 2370–2383. 11675413 10.1681/ASN.V12112370

[B33] Gotoh M. Monden M. Okamura J. Mori T. Shima K. (1989). Insulin and glucagon secretion after pancreatectomies. Correlation of secretion and hormonal contents of remaining pancreas. *Diabetes* 38 861–867. 10.2337/diab.38.7.861 2661285

[B34] Guder W. Hepp K. D. Wieland O. (1970). The catabolic action of glucagon in rat liver. The influence of age, nutritional state and adrenal function on the effect of glucagon on lysosomal N-acetyl-beta, D-glucosaminidase. *Biochim. Biophys. Acta* 222 593–605. 10.1016/0304-4165(70)90185-6 4322197

[B35] Gupta N. A. Mells J. Dunham R. M. Grakoui A. Handy J. Saxena N. K. (2010). Glucagon-like peptide-1 receptor is present on human hepatocytes and has a direct role in decreasing hepatic steatosis in vitro by modulating elements of the insulin signaling pathway. *Hepatology* 51 1584–1592. 10.1002/hep.23569 20225248 PMC2862093

[B36] Han V. K. Hynes M. A. Jin C. Towle A. C. Lauder J. M. Lund P. K. (1986). Cellular localization of proglucagon/glucagon-like peptide I messenger RNAs in rat brain. *J. Neurosci. Res.* 16 97–107. 10.1002/jnr.490160110 2427741

[B37] He B. Liu L. Yu C. Wang Y. Han P. (2015). Roux-en-Y gastric bypass reduces lipid overaccumulation in liver by upregulating hepatic autophagy in obese diabetic rats. *Obes. Surg.* 25 109–118. 10.1007/s11695-014-1342-7 24993523

[B38] He Q. Sha S. Sun L. Zhang J. Dong M. (2016). GLP-1 analogue improves hepatic lipid accumulation by inducing autophagy via AMPK/mTOR pathway. *Biochem. Biophys. Res. Commun.* 476 196–203. 10.1016/j.bbrc.2016.05.086 27208776

[B39] Hollande C. Boussier J. Ziai J. Nozawa T. Bondet V. Phung W. (2019). Inhibition of the dipeptidyl peptidase DPP4 (CD26) reveals IL-33-dependent eosinophil-mediated control of tumor growth. *Nat. Immunol.* 20 257–264. 10.1038/s41590-019-0321-5 30778250

[B40] Holst J. J. Bersani M. Johnsen A. H. Kofod H. Hartmann B. Orskov C. (1994). Proglucagon processing in porcine and human pancreas. *J. Biol. Chem.* 269 18827–18833. 8034635

[B41] Holst J. J. Pedersen J. H. Baldissera F. Stadil F. (1983). Circulating glucagon after total pancreatectomy in man. *Diabetologia* 25 396–399. 10.1007/bf00282517 6653943

[B42] Horowitz M. Cook D. J. Collins P. J. Harding P. E. Hooper M. J. Walsh J. F. (1982). Measurement of gastric emptying after gastric bypass surgery using radionuclides. *Br. J. Surg.* 69 655–657. 10.1002/bjs.1800691108 7127049

[B43] Hynes R. O. (2002). Integrins: bidirectional, allosteric signaling machines. *Cell* 110 673–687. 12297042 10.1016/s0092-8674(02)00971-6

[B44] Ichimura Y. Waguri S. Sou Y. S. Kageyama S. Hasegawa J. Ishimura R. (2013). Phosphorylation of p62 activates the Keap1-Nrf2 pathway during selective autophagy. *Mol. Cell* 51 618–631. 10.1016/j.molcel.2013.08.003 24011591

[B45] Itou M. Kawaguchi T. Taniguchi E. Sata M. (2013). Dipeptidyl peptidase-4: a key player in chronic liver disease. *World J. Gastroenterol.* 19 2298–2306. 10.3748/wjg.v19.i15.2298 23613622 PMC3631980

[B46] Jain A. Lamark T. Sjottem E. Larsen K. B. Awuh J. A. Overvatn A. (2010). p62/SQSTM1 is a target gene for transcription factor NRF2 and creates a positive feedback loop by inducing antioxidant response element-driven gene transcription. *J. Biol. Chem.* 285 22576–22591. 10.1074/jbc.M110.118976 20452972 PMC2903417

[B47] Jin S. L. Han V. K. Simmons J. G. Towle A. C. Lauder J. M. Lund P. K. (1988). Distribution of glucagonlike peptide I (GLP-I), glucagon, and glicentin in the rat brain: an immunocytochemical study. *J. Comp. Neurol.* 271 519–532. 10.1002/cne.902710405 3385016

[B48] Jin T. (2008). Mechanisms underlying proglucagon gene expression. *J. Endocrinol.* 198 17–28. 10.1677/JOE-08-0085 18577568

[B49] Jin T. Weng J. (2016). Hepatic functions of GLP-1 and its based drugs: current disputes and perspectives. *Am. J. Physiol. Endocrinol. Metab.* 311 E620–E627. 10.1152/ajpendo.00069.2016 27507553

[B50] Kahne T. Lendeckel U. Wrenger S. Neubert K. Ansorge S. Reinhold D. (1999). Dipeptidyl peptidase IV: a cell surface peptidase involved in regulating T cell growth (review). *Int. J. Mol. Med.* 4 3–15. 10373631 10.3892/ijmm.4.1.3

[B51] Kameoka J. Tanaka T. Nojima Y. Schlossman S. F. Morimoto C. (1993). Direct association of adenosine deaminase with a T cell activation antigen, CD26. *Science* 261 466–469. 10.1126/science.8101391 8101391

[B52] Kanasaki K. (2016). The pathological significance of dipeptidyl peptidase-4 in endothelial cell homeostasis and kidney fibrosis. *Diabetol. Int.* 7 212–220. 10.1007/s13340-016-0281-z 30603266 PMC6224988

[B53] Karesen R. Tronier B. Aune S. (1980). Immunoreactive glucagon and insulin C-peptide in man after resection of the pancreas and total pancreatectomy. *Am. J. Surg.* 140 272–276. 10.1016/0002-9610(80)90021-5 6996505

[B54] Kaspar J. W. Niture S. K. Jaiswal A. K. (2009). Nrf2:INrf2 (Keap1) signaling in oxidative stress. *Free Radic. Biol. Med.* 47 1304–1309. 10.1016/j.freeradbiomed.2009.07.035 19666107 PMC2763938

[B55] Komatsu M. Kurokawa H. Waguri S. Taguchi K. Kobayashi A. Ichimura Y. (2010). The selective autophagy substrate p62 activates the stress responsive transcription factor Nrf2 through inactivation of Keap1. *Nat. Cell Biol.* 12 213–223. 10.1038/ncb2021 20173742

[B56] Kotler D. P. Sherman D. Bloom S. R. Holt P. R. (1985). Malnutrition after gastric surgery. Association with exaggerated distal intestinal hormone release. *Dig. Dis. Sci.* 30 193–199. 10.1007/bf01347882 3971831

[B57] Kroller-Schon S. Knorr M. Hausding M. Oelze M. Schuff A. Schell R. (2012). Glucose-independent improvement of vascular dysfunction in experimental sepsis by dipeptidyl-peptidase 4 inhibition. *Cardiovasc. Res.* 96 140–149. 10.1093/cvr/cvs246 22843705

[B58] Lambeir A. M. Durinx C. Scharpe S. De Meester I. (2003). Dipeptidyl-peptidase IV from bench to bedside: an update on structural properties, functions, and clinical aspects of the enzyme DPP IV. *Crit. Rev. Clin. Lab. Sci.* 40 209–294. 10.1080/713609354 12892317

[B59] Larsen P. J. Tang-Christensen M. Holst J. J. Orskov C. (1997). Distribution of glucagon-like peptide-1 and other preproglucagon-derived peptides in the rat hypothalamus and brainstem. *Neuroscience* 77 257–270. 10.1016/s0306-4522(96)00434-4 9044391

[B60] Lau A. Wang X. J. Zhao F. Villeneuve N. F. Wu T. Jiang T. (2010). A noncanonical mechanism of Nrf2 activation by autophagy deficiency: direct interaction between Keap1 and p62. *Mol. Cell. Biol.* 30 3275–3285. 10.1128/MCB.00248-10 20421418 PMC2897585

[B61] Lim S. W. Jin L. Jin J. Yang C. W. (2016). Effect of exendin-4 on autophagy clearance in beta cell of rats with tacrolimus-induced diabetes mellitus. *Sci. Rep.* 6:29921. 10.1038/srep29921 27436514 PMC4951772

[B62] Liu L. Liu J. Yu X. (2016). Dipeptidyl peptidase-4 inhibitor MK-626 restores insulin secretion through enhancing autophagy in high fat diet-induced mice. *Biochem. Biophys. Res. Commun.* 470 516–520. 10.1016/j.bbrc.2016.01.116 26802468

[B63] Liu Z. Stanojevic V. Brindamour L. J. Habener J. F. (2012). GLP1-derived nonapeptide GLP1(28-36)amide protects pancreatic beta-cells from glucolipotoxicity. *J. Endocrinol.* 213 143–154. 10.1530/JOE-11-0328 22414687 PMC4096040

[B64] Lopez-Otin C. Matrisian L. M. (2007). Emerging roles of proteases in tumour suppression. *Nat. Rev. Cancer* 7 800–808. 10.1038/nrc2228 17851543

[B65] Lu G. Hu Y. Wang Q. Qi J. Gao F. Li Y. (2013). Molecular basis of binding between novel human coronavirus MERS-CoV and its receptor CD26. *Nature* 500 227–231. 10.1038/nature12328 23831647 PMC7095341

[B66] Lund A. Bagger J. I. Wewer Albrechtsen N. J. Christensen M. Grondahl M. Hartmann B. (2016). Evidence of extrapancreatic glucagon secretion in man. *Diabetes Metab. Res. Rev.* 65 585–597. 10.2337/db15-1541 26672094

[B67] Luo B. H. Carman C. V. Springer T. A. (2007). Structural basis of integrin regulation and signaling. *Annu. Rev. Immunol.* 25 619–647. 10.1146/annurev.immunol.25.022106.141618 17201681 PMC1952532

[B68] Ma Q. (2013). Role of nrf2 in oxidative stress and toxicity. *Annu. Rev. Pharmacol. Toxicol.* 53 401–426. 10.1146/annurev-pharmtox-011112-140320 23294312 PMC4680839

[B69] Maiztegui B. Boggio V. Roman C. L. Flores L. E. Zotto H. D. Ropolo A. (2017). VMP1-related autophagy induced by a fructose-rich diet in beta-cells: its prevention by incretins. *Clin. Sci.* 131 673–687. 10.1042/CS20170010 28188238

[B70] Marso S. P. Daniels G. H. Brown-Frandsen K. Kristensen P. Mann J. F. Nauck M. A. (2016). Liraglutide and cardiovascular outcomes in type 2 diabetes. *N. Engl. J. Med.* 375 311–322. 27295427 10.1056/NEJMoa1603827 PMC4985288

[B71] Marso S. P. Holst A. G. Vilsboll T. (2017). Semaglutide and cardiovascular outcomes in patients with type 2 diabetes. *N. Engl. J. Med.* 376 891–892. 10.1056/NEJMc1615712 28249135

[B72] Matsuyama T. Foa P. P. (1974). Plasma glucose, insulin, pancreatic, and enteroglucagon levels in normal and depancreatized dogs. *Proc. Soc. Exp. Biol. Med.* 147 97–102. 10.3181/00379727-147-38288 4438349

[B73] Mentis N. Vardarli I. Kothe L. D. Holst J. J. Deacon C. F. Theodorakis M. (2011). GIP does not potentiate the antidiabetic effects of GLP-1 in hyperglycemic patients with type 2 diabetes. *Diabetes Metab. Res. Rev.* 60 1270–1276. 10.2337/db10-1332 21330636 PMC3064100

[B74] Moellmann J. Klinkhammer B. M. Onstein J. Stohr R. Jankowski V. Jankowski J. (2018). Glucagon-like peptide 1 and its cleavage products are renoprotective in murine diabetic nephropathy. *Diabetes Metab. Res. Rev.* 67 2410–2419. 10.2337/db17-1212 30104246

[B75] Mortimore G. E. Poso A. R. (1987). Intracellular protein catabolism and its control during nutrient deprivation and supply. *Annu. Rev. Nutr.* 7 539–564. 10.1146/annurev.nutr.7.1.539 3300746

[B76] Muller T. D. Finan B. Clemmensen C. DiMarchi R. D. Tschop M. H. (2017). The new biology and pharmacology of glucagon. *Physiol. Rev.* 97 721–766. 10.1152/physrev.00025.2016 28275047

[B77] Muller W. A. Girardier L. Seydoux J. Berger M. Renold A. E. Vranic M. (1978). Extrapancreatic glucagon and glucagonlike immunoreactivity in depancreatized dogs. A quantitative assessment of secretion rates and anatomical delineation of sources. *J. Clin. Invest.* 62 124–132. 10.1172/jci109096 659625 PMC371745

[B78] Murase H. Kuno A. Miki T. Tanno M. Yano T. Kouzu H. (2015). Inhibition of DPP-4 reduces acute mortality after myocardial infarction with restoration of autophagic response in type 2 diabetic rats. *Cardiovasc. Diabetol.* 14:103. 10.1186/s12933-015-0264-6 26259714 PMC4531441

[B79] Novikoff A. B. Beaufay H. De Duve C. (1956). Electron microscopy of lysosomerich fractions from rat liver. *J. Biophys. Biochem. Cyto.l* 2 179–184. 10.1083/jcb.2.4.179 13357540 PMC2229688

[B80] Oh Y. S. Jun H. S. (2017). Effects of glucagon-like peptide-1 on oxidative stress and Nrf2 signaling. *Int. J. Mol. Sci.* 19:E26. 10.3390/ijms19010026 29271910 PMC5795977

[B81] Ohtsuka K. Nimura Y. Yasui K. (1986). Paradoxical elevations of plasma glucagon levels in patients after pancreatectomy or gastrectomy. *Jpn. J. Surg.* 16 1–7. 10.1007/bf02471062 3959356

[B82] O’Malley T. J. Fava G. E. Zhang Y. Fonseca V. A. Wu H. (2014). Progressive change of intra-islet GLP-1 production during diabetes development. *Diabetes Metab. Res. Rev.* 30 661–668. 10.1002/dmrr.2534 24510483 PMC4126896

[B83] Park K. S. Ki C. S. Lee N. Y. (2015). Isolation and identification of clostridium difficile using ChromID *C. difficile* medium combined with gram staining and pro disc testing: a proposal for a simple culture process. *Ann. Lab. Med.* 35 404–409. 10.3343/alm.2015.35.4.404 26131411 PMC4446578

[B84] Plow E. F. Haas T. A. Zhang L. Loftus J. Smith J. W. (2000). Ligand binding to integrins. *J. Biol. Chem.* 275 21785–21788. 10801897 10.1074/jbc.R000003200

[B85] Polonsky K. S. Herold K. C. Gilden J. L. Bergenstal R. M. Fang V. S. Moossa A. R. (1984). Glucose counterregulation in patients after pancreatectomy. Comparison with other clinical forms of diabetes. *Diabetes* 33 1112–1119. 10.2337/diab.33.11.1112 6389228

[B86] Pozzi A. Zent R. (2003). Integrins: sensors of extracellular matrix and modulators of cell function. *Nephron Exp. Nephrol.* 94 e77–e84. 10.1159/000072025 12902617

[B87] Pozzi A. Zent R. (2011). Extracellular matrix receptors in branched organs. *Curr. Opin. Cell Biol.* 23 547–553. 10.1016/j.ceb.2011.04.003 21561755 PMC3181278

[B88] Quercia I. Dutia R. Kotler D. P. Belsley S. Laferrere B. (2014). Gastrointestinal changes after bariatric surgery. *Diabetes Metab.* 40 87–94. 10.1016/j.diabet.2013.11.003 24359701 PMC4391395

[B89] Ratnikov B. I. Partridge A. W. Ginsberg M. H. (2005). Integrin activation by talin. *J. Thromb. Haemost.* 3 1783–1790. 16102045 10.1111/j.1538-7836.2005.01362.x

[B90] Rosenstock J. Perkovic V. Johansen O. E. Cooper M. E. Kahn S. E. Marx N. (2019). Effect of linagliptin vs placebo on major cardiovascular events in adults with type 2 diabetes and high cardiovascular and renal risk: the carmelina randomized clinical trial. *JAMA* 321 69–79. 10.1001/jama.2018.18269 30418475 PMC6583576

[B91] Rowlands J. Heng J. Newsholme P. Carlessi R. (2018). Pleiotropic effects of GLP-1 and analogs on cell signaling, metabolism, and function. *Front. Endocrinol.* 9:672 10.3389/fendo.2018.00672 PMC6266510 30532733

[B92] Sato T. Yamochi T. Yamochi T. Aytac U. Ohnuma K. McKee K. S. (2005). CD26 regulates p38 mitogen-activated protein kinase-dependent phosphorylation of integrin beta1, adhesion to extracellular matrix, and tumorigenicity of T-anaplastic large cell lymphoma Karpas 299. *Cancer Res.* 65 6950–6956. 10.1158/0008-5472.can-05-0647 16061680

[B93] Sharma S. Mells J. E. Fu P. P. Saxena N. K. Anania F. A. (2011). GLP-1 analogs reduce hepatocyte steatosis and improve survival by enhancing the unfolded protein response and promoting macroautophagy. *PLoS One* 6:e25269. 10.1371/journal.pone.0025269 21957486 PMC3177901

[B94] Shelburne J. D. Arstila A. U. Trump B. F. (1973). Studies on cellular autophagocytosis. The relationship of autophagocytosis to protein synthesis and to energy metabolism in rat liver and flounder kidney tubules in vitro. *Am. J. Pathol.* 73 641–670. 4767257 PMC1904080

[B95] Shi S. Srivastava S. P. Kanasaki M. He J. Kitada M. Nagai T. (2015). Interactions of DPP-4 and integrin beta1 influences endothelial-to-mesenchymal transition. *Kidney Int.* 88 479–489. 10.1038/ki.2015.103 25830763

[B96] Singh K. K. Lovren F. Pan Y. Quan A. Ramadan A. Matkar P. N. (2015). The essential autophagy gene ATG7 modulates organ fibrosis via regulation of endothelial-to-mesenchymal transition. *J. Biol. Chem.* 290 2547–2559. 10.1074/jbc.M114.604603 25527499 PMC4317030

[B97] Soussi H. Clement K. Dugail I. (2016). Adipose tissue autophagy status in obesity: expression and flux–two faces of the picture. *Autophagy* 12 588–589. 10.1080/15548627.2015.1106667 26565777 PMC4835957

[B98] Sudo T. Suzuki T. Tobe T. (1980). Changes in plasma glucagon after total pancreatectomy in man. *Gastroenterol. Jpn.* 15 464–468. 10.1007/bf02773909 7002702

[B99] Sutherland E. W. De Duve C. (1948). Origin and distribution of the hyperglycemic-glycogenolytic factor of the pancreas. *J. Biol. Chem.* 175 663–674. 18880761

[B100] Svane M. S. Bojsen-Moller K. N. Martinussen C. Dirksen C. Madsen J. L. Reitelseder S. (2019). Postprandial nutrient handling and gastrointestinal secretion of hormones after roux-en-Y gastric Bypass vs Sleeve gastrectomy. *Gastroenterology* 156 1627–1641.e1. 10.1053/j.gastro.2019.01.262 30742833

[B101] Taguchi K. Fujikawa N. Komatsu M. Ishii T. Unno M. Akaike T. (2012). Keap1 degradation by autophagy for the maintenance of redox homeostasis. *Proc. Natl. Acad. Sci. U.S.A.* 109 13561–13566. 10.1073/pnas.1121572109 22872865 PMC3427110

[B102] Talukdar S. Pradhan A. K. Bhoopathi P. Shen X. N. August L. A. Windle J. J. (2018). Regulation of protective autophagy in anoikis-resistant glioma stem cells by SDCBP/MDA-9/Syntenin. *Autophagy* 14 1845–1846. 10.1080/15548627.2018.1502564 30118375 PMC6135626

[B103] Tanjoh K. Tomita R. Fukuzawa M. Hayashi N. (2003). Peculiar glucagon processing in the intestine is the genesis of the paradoxical rise of serum pancreatic glucagon in patients after total pancreatectomy. *Hepatogastroenterology* 50 535–540. 12749267

[B104] Tomas E. Stanojevic V. Habener J. F. (2010). GLP-1 (9-36) amide metabolite suppression of glucose production in isolated mouse hepatocytes. *Horm. Metab. Res.* 42 657–662. 10.1055/s-0030-1253421 20645222

[B105] Tucker J. D. Dhanvantari S. Brubaker P. L. (1996). Proglucagon processing in islet and intestinal cell lines. *Regul. Pept.* 62 29–35. 10.1016/0167-0115(95)00167-0 8738879

[B106] Ueno T. Komatsu M. (2017). Autophagy in the liver: functions in health and disease. *Nat. Rev. Gastroenterol. Hepatol.* 14 170–184. 10.1038/nrgastro.2016.185 28053338

[B107] Unger R. H. Ketterer H. Eisentraut A. M. (1966). Distribution of immunoassayable glucagon in gastrointestinal tissues. *Metabolism* 15 865–867. 10.1016/0026-0495(66)90156-9 5923522

[B108] Villanueva M. L. Hedo J. A. Marco J. (1976). Plasma glucagon immunoreactivity in a totally pancreatectomized patient. *Diabetologia* 12 613–616. 10.1007/bf01220639 1001850

[B109] Vlahakis A. Debnath J. (2017). The interconnections between autophagy and integrin-mediated cell adhesion. *J. Mol. Biol.* 429 515–530. 10.1016/j.jmb.2016.11.027 27932295 PMC5276719

[B110] Vrang N. Hansen M. Larsen P. J. Tang-Christensen M. (2007). Characterization of brainstem preproglucagon projections to the paraventricular and dorsomedial hypothalamic nuclei. *Brain Res.* 1149 118–126. 10.1016/j.brainres.2007.02.043 17433266

[B111] Vranic M. Pek S. Kawamori R. (1974). Increased “glucagon immunoreactivity” in plasma of totally depancreatized dogs. *Diabetes Metab. Res. Rev.* 23 905–912. 10.2337/diab.23.11.905 4430418

[B112] Wewer Albrechtsen N. J. Hartmann B. Veedfald S. Windelov J. A. Plamboeck A. Bojsen-Moller K. N. (2014). Hyperglucagonaemia analysed by glucagon sandwich ELISA: nonspecific interference or truly elevated levels? *Diabetologia* 57 1919–1926. 10.1007/s00125-014-3283-z 24891019

[B113] Yang F. Takagaki Y. Yoshitomi Y. Ikeda T. Li J. Kitada M. (2019). Inhibition of dipeptidyl peptidase-4 accelerates epithelial-mesenchymal transition and breast cancer metastasis via the CXCL12/CXCR4/mTOR axis. *Cancer Res.* 79 735–746. 10.1158/0008-5472.CAN-18-0620 30584072

[B114] Yang J. Zheng Z. Yan X. Li X. Liu Z. Ma Z. (2013). Integration of autophagy and anoikis resistance in solid tumors. *Anat. Rec.* 296 1501–1508. 10.1002/ar.22769 23963853

[B115] Yang Y. Cuevas S. Yang S. Villar V. A. Escano C. Asico L. (2014). Sestrin2 decreases renal oxidative stress, lowers blood pressure, and mediates dopamine D2 receptor-induced inhibition of reactive oxygen species production. *Hypertension* 64 825–832. 10.1161/HYPERTENSIONAHA.114.03840 25024286 PMC4162832

[B116] Yasui K. (1983). Effects of total pancreatectomy on the secretion of gut glucagon in humans. *Jpn. J. Surg.* 13 122–129. 10.1007/bf02469532 6350663

[B117] Ye H. Adane B. Khan N. Alexeev E. Nusbacher N. Minhajuddin M. (2018). Subversion of systemic glucose metabolism as a mechanism to support the growth of leukemia cells. *Cancer Cell* 34 659–673.e6. 10.1016/j.ccell.2018.08.016 30270124 PMC6177322

[B118] Yi F. Sun J. Lim G. E. Fantus I. G. Brubaker P. L. Jin T. (2008). Cross talk between the insulin and Wnt signaling pathways: evidence from intestinal endocrine L cells. *Endocrinology* 149 2341–2351. 10.1210/en.2007-1142 18258680

[B119] Zheng W. Zhou J. Song S. Kong W. Xia W. Chen L. (2018). Dipeptidyl-peptidase 4 inhibitor sitagliptin ameliorates hepatic insulin resistance by modulating inflammation and autophagy in ob/ob mice. *Int. J. Endocrinol.* 2018:8309723. 10.1155/2018/8309723 30123267 PMC6079465

[B120] Zhuge F. Ni Y. Nagashimada M. Nagata N. Xu L. Mukaida N. (2016). DPP-4 inhibition by linagliptin attenuates obesity-related inflammation and insulin resistance by regulating M1/M2 macrophage polarization. *Diabetes Metab. Res. Rev.* 65 2966–2979. 10.2337/db16-0317 27445264

[B121] Zummo F. P. Cullen K. S. Honkanen-Scott M. Shaw J. A. M. Lovat P. E. Arden C. (2017). Glucagon-like peptide 1 protects pancreatic beta-cells from death by increasing autophagic flux and restoring lysosomal function. *Diabetes Metab. Res. Rev.* 66 1272–1285. 10.2337/db16-1009 28232493

